# Case Report: Pediatric MOG/NMDAR overlap syndrome with delayed cortical MRI evolution and incidental venous sinus hypoplasia

**DOI:** 10.3389/fimmu.2026.1836078

**Published:** 2026-07-03

**Authors:** Wenqing Cao, Zezhen Chen, Mei Liu, Jin Wu

**Affiliations:** The Second Clinical Medical College of Nanjing Medical University, Nanjing, China

**Keywords:** anti-NMDAR encephalitis, cortical encephalitis, immunotherapy, longitudinal follow-up, MNOS, MOGAD, overlapping antibody syndrome, pediatric

## Abstract

**Background:**

Pediatric overlap of myelin oligodendrocyte glycoprotein antibody-associated disease (MOGAD) and anti-N-methyl-D-aspartate receptor encephalitis (MNOS) is uncommon. At onset, such cases may resemble infectious or vascular disorders, leading to premature diagnostic closure and delayed immunotherapy. Most published reports emphasize antibody coexistence itself, whereas fewer describe how misleading early clinical or imaging clues can redirect the diagnostic pathway and how longitudinal multimodal reassessment can correct the initial working diagnosis.

**Case presentation:**

A 16-year-old girl presented with a 20-day history of progressive headache, transient focal deficits, meningeal irritation, and inflammatory CSF abnormalities. Relative-timeline reassessment showed inflammatory CSF on Day 4, a left parieto-occipital cortical-sulcal FLAIR abnormality by Week 2, MRV findings favoring unilateral transverse-sigmoid sinus hypoplasia/low-flow change rather than active thrombosis at Month 1, and relapse with seizure and a new right occipital cortical-sulcal lesion at Month 3. Paired CSF/serum antibody testing revealed CSF anti-NMDAR-IgG positivity together with CSF/serum MOG-IgG positivity, supporting a final diagnosis of MNOS-associated MOGAD cortical encephalitis within the FLAMES/UCCE spectrum. The patient improved after high-dose corticosteroids and intravenous immunoglobulin, with no further documented seizures during follow-up.

**Conclusions:**

This case illustrates that MNOS-related FLAMES/UCCE-spectrum cortical encephalitis may initially raise concern for CVST or viral meningoencephalitis when early parenchymal MRI is nondiagnostic and venous imaging abnormalities coexist. Diagnostic adjudication should not rely on a single imaging clue or isolated antibody result, but on integrated reassessment of the clinical phenotype, serial MRI evolution, CSF inflammatory features, and standardized antibody testing. Persistent serum MOG-IgG may support concern for a relapsing-prone disease course, but a low-positive titer alone should not be used as a stand-alone marker of active relapse without clinicoradiological support.

## Introduction

MNOS is rare in children and may be difficult to recognize when early neuroimaging is normal, nonspecific, or delayed relative to clinical symptoms (1–4). At onset, it may resemble infectious meningoencephalitis or CVST, particularly when headache, meningeal signs, inflammatory CSF abnormalities, and venous imaging abnormalities coexist. In our patient, outside-hospital MRV showed unilateral non-visualization of the left transverse-sigmoid sinus and internal jugular vein, but the initial parenchymal MRI report showed no venous infarction or other secondary signs of active CVST, and no clinical signs of intracranial hypertension were documented before lumbar puncture. Longitudinal review therefore favored an incidental hypoplastic or low-flow venous variant, while sequential MRI revealed delayed cortical-sulcal lesions. This case highlights the value of longitudinal clinicoradiological reassessment in distinguishing MNOS-related MOGAD cortical encephalitis within the FLAMES/UCCE spectrum from infectious and vascular mimics (3, 5–7, 9, 10).

## Case presentation

**Figure 1:**
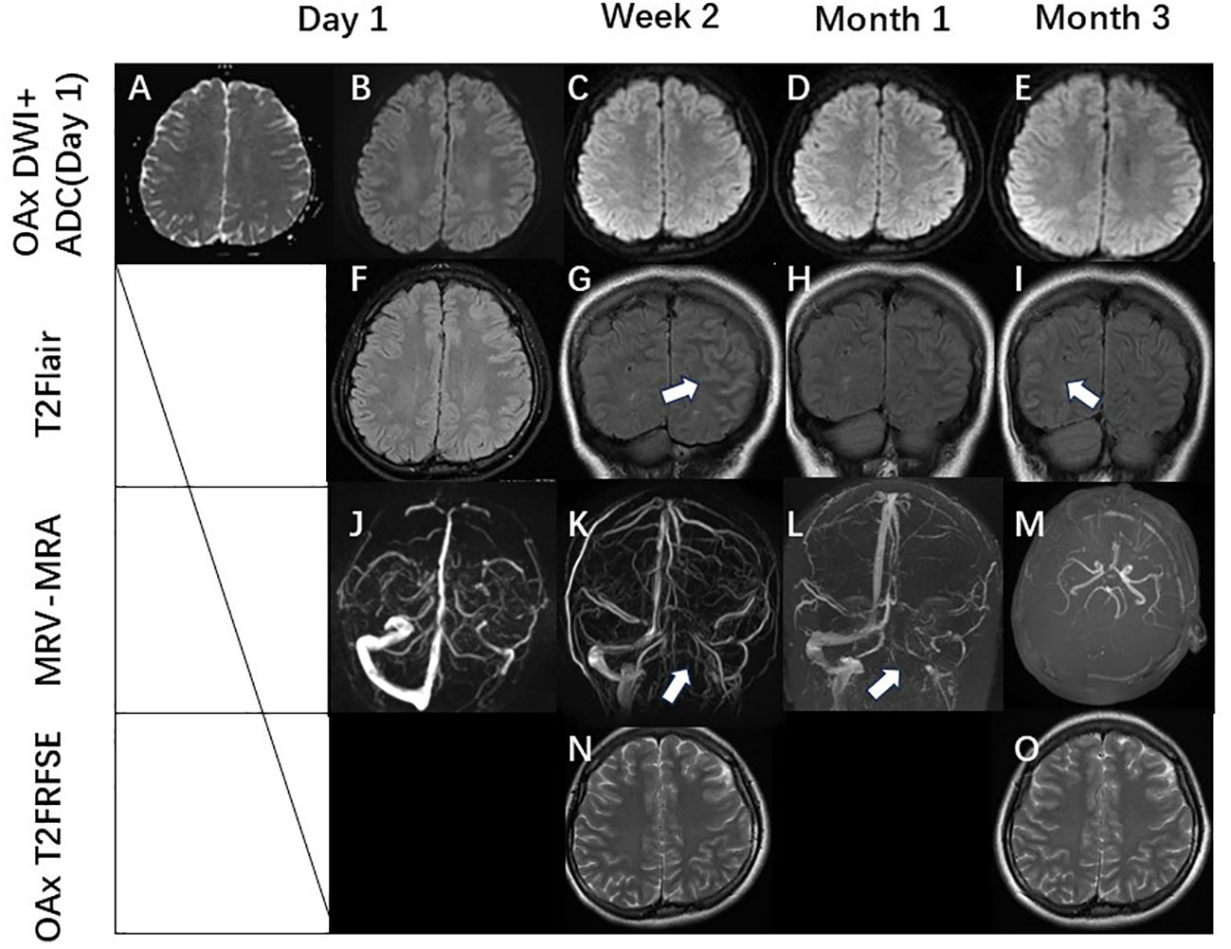
Serial MRI/MRV evolution during diagnostic reassessment. Representative images are shown using de-identified relative timepoints **(A–O)**. **(A)** Day 1 ADC; **(B)** Day 1 DWI; **(C)** Week 2 DWI; **(D)** Month 1 DWI; **(E)** Month 3 DWI; **(F)** Day 1 T2Flair; **(G)** Week 2 T2Flair; **(H)** Month 1 T2Flair; **(I)** Month 3 T2Flair; **(J)** Day 1 MRV-MIP; **(K)** Week 2 MRV; **(L)** Month 1 MRV; **(M)** Month 3 MRA; **(N)** Week 2 T2-FRFSE **(O)** Month 3 T2-FRFSE At Day 1, outside-hospital ADC **(A)**, DWI **(B)**,T2-FLAIR **(F)**, and MRV-MIP **(J)** images are shown. The parenchymal sequences did not demonstrate definite restricted diffusion, venous-territory infarction, hemorrhagic venous infarction, mass effect, or other secondary signs of venous hypertension, whereas MRV-MIP **(J)** showed left transverse-sigmoid sinus/internal jugular venous non-visualization/asymmetry. At Week 2, oblique coronal T2-FLAIR **(G)** demonstrated a left parieto-occipital cortical-sulcal hyperintensity, with corresponding T2-FRFSE **(N)** and DWI **(C)** images shown for anatomical and diffusion-weighted correlation. At Month 1, follow-up T2-FLAIR **(H)** showed no progressive venous-territory parenchymal injury, while MRV **(L)** showed persistent left-sided venous asymmetry, supporting reinterpretation as a hypoplastic or low-flow variant rather than active progressive CVST. At Month 3, relapse MRI showed a new right occipital cortical-sulcal T2-FLAIR **(I)** hyperintensity, with corresponding T2-FRFSE **(O)** and DWI **(E)** images, indicating a relapsing cortical encephalitic pattern with changing laterality. Post-gadolinium T1-weighted images were not available for the displayed timepoints. Arrows indicate cortical-sulcal lesions; arrowheads indicate venous asymmetry.

A 16-year-old girl with a history of Helicobacter pylori–treated gastric ulcer, recurrent oral ulcers since childhood, and prior appendectomy, but no seizures or known inflammatory CNS disease, was admitted after 20 days of progressive headache with acute worsening over 1 day. Before admission, she developed transient right-sided weakness, right upper-limb involuntary movements, and speech disturbance. Outside-hospital non-contrast brain MRI/MRV showed no obvious parenchymal lesion but demonstrated non-visualization of the left transverse sinus, sigmoid sinus, and internal jugular vein (**Figures 1A, B, F, J**, Day 1), prompting a presumptive diagnosis of active CVST and empirical LMWH during transfer. However, upon subsequent retrieval and detailed retrospective review of the original outside-hospital images at our institution—including diffusion-weighted imaging (DWI), apparent diffusion coefficient (ADC), T2-fluid-attenuated inversion recovery (T2-FLAIR), and MRV-MIP sequences—no definite parenchymal lesion, restricted diffusion, venous-territory infarction, hemorrhagic venous infarction, mass effect, or other secondary signs of venous hypertension were identified on the available parenchymal sequences (**Figures 1A, B, F, J**, Day 1). The MRV-MIP image confirmed non-visualization and asymmetry of the left transverse-sigmoid sinus and internal jugular venous outflow pathway (**Figure 1J**, Day 1). This imaging suspicion was discordant with the clinical and laboratory profile. No papilledema, diplopia, sixth-nerve palsy, transient visual obscurations, or pulsatile tinnitus was documented before lumbar puncture, and plasma D-dimer levels remained normal. Lumbar puncture instead showed marked lymphocyte/monocyte-predominant CSF pleocytosis. Without corroborating parenchymal injury on DWI/ADC or T2-FLAIR, isolated venous non-visualization was considered insufficient to establish active CVST and later favored a hypoplastic or low-flow variant. During transfer, some symptoms improved spontaneously, but right upper-limb weakness, numbness, and gait weakness persisted. On admission, examination showed right upper-limb weakness of approximately 4/5, hypoalgesia, neck stiffness, and a positive Brudzinski sign. LMWH was continued while vascular, infectious, and immune-mediated etiologies were investigated.

**Figure 2:**
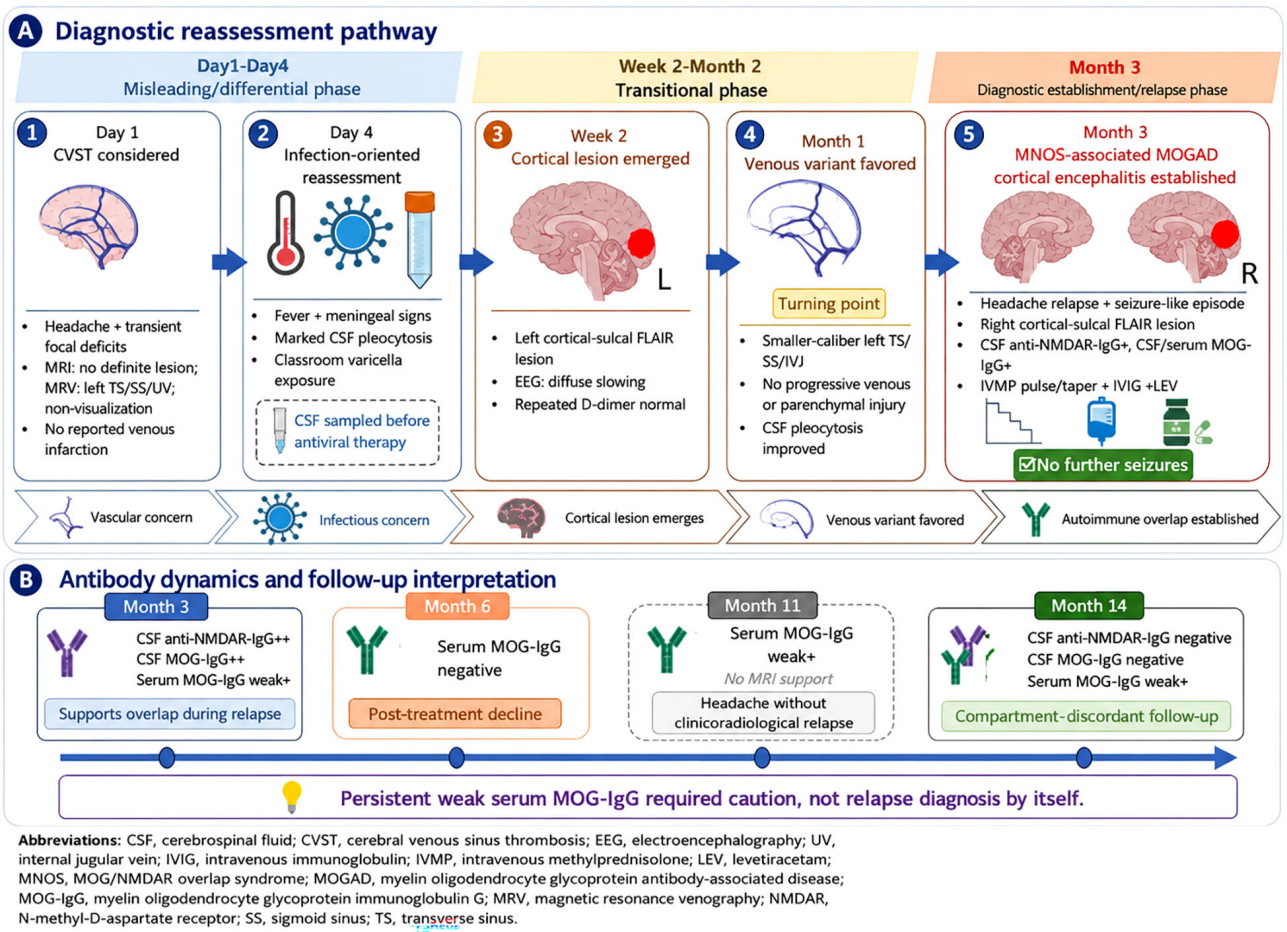
Integrated diagnostic reassessment pathway and antibody dynamics in pediatric MOG/NMDAR overlap syndrome. **(A)** The diagnostic pathway is shown using de-identified relative timepoints. The early phase was dominated by vascular and infection-oriented differentials because of headache, transient focal deficits, meningeal signs, CSF pleocytosis, and left transverse-sigmoid sinus/internal jugular vein non-visualization on MRV. Sequential reassessment showed the emergence of unilateral cortical-sulcal FLAIR lesions, stable venous asymmetry favoring a hypoplastic or low-flow variant, and later contralateral cortical involvement with paired CSF/serum autoantibody positivity, supporting MNOS-associated MOGAD cortical encephalitis. **(B)** Antibody dynamics during follow-up. Persistent weak serum MOG-IgG was interpreted as supportive of relapse-risk awareness but not as sufficient evidence of active relapse without concordant clinical or MRI findings. Relative timing is used to preserve patient anonymity.

During hospitalization, the patient continued to have headache and developed a low-grade fever, with a maximum temperature of 37.7 °C. Meningeal signs persisted. A lumbar puncture performed 4 days after admission revealed an opening pressure of 210 mmH_2_O and a CSF white blood cell count of approximately 241 cells/μL, predominantly lymphocytes/monocytes. India ink staining, Gram staining, acid-fast staining, and CSF culture were all negative, while CSF biochemistry remained compatible with an inflammatory process. Family members reported a recent classroom varicella outbreak, and the patient had not received varicella vaccination. In the context of headache, low-grade fever, meningeal signs, and lymphocyte/monocyte-predominant CSF pleocytosis, a herpesvirus-related meningoencephalitic process was considered clinically plausible during the first admission. DNA-based pathogen high-throughput sequencing was negative, and specific PCR for HSV/VZV was not available. However, in the context of an ongoing classroom varicella outbreak and an unvaccinated patient, VZV encephalitis remained a primary clinical concern. Empirical anti-herpesvirus treatment was therefore initiated with ganciclovir — the only intravenous antiviral agent with anti-herpesvirus activity available at our institution at that time — together with intracranial pressure−lowering therapy. Taken together, the early infectious workup lowered the likelihood of an active DNA-pathogen CNS infection at the sampled timepoint, but did not microbiologically confirm or definitively exclude a herpesvirus-related meningoencephalitic or post-infectious process.

Over the following days, the patient began to improve before corticosteroid escalation, although headache and meningeal irritation persisted. Because diagnostic uncertainty remained and neuro-Behçet disease was still considered, rheumatology consultation was obtained, and intravenous methylprednisolone sodium succinate 40 mg once daily with gastric protection was added. Ceftriaxone was briefly administered because bacterial infection had not been fully excluded, while LMWH was reduced as active CVST became less likely. Headache then improved substantially, and the initial right-sided weakness and sensory symptoms gradually resolved. Week 2 EEG showed mild-to-moderate diffuse slowing, with left-predominant bilateral frontal delta activity and no extreme delta brush. On Day 11, scattered pruritic pinpoint papules appeared on the hands and abdomen; dermatology favored a nonspecific viral exanthem without pathogen-specific morphology. Week 2 MRI/MRV showed a left parieto-occipital sulcal T2-FLAIR hyperintensity (**Figure 1G**, Week 2) and persistent left transverse-sigmoid sinus/internal jugular vein non-visualization (**Figure 1K**, Week 2). At this stage, the case remained diagnostically ambiguous: the inflammatory CSF profile and rash supported an infection-like presentation, whereas the evolving cortical/sulcal MRI abnormality and repeatedly normal D-dimer values were less consistent with a straightforward thrombotic process.

At Month 1, the patient was readmitted for worsening intermittent headache over 2 days, without definite focal deficits. Repeat MRI showed no definite parenchymal lesion (**Figures 1D, H**, Month 1). MRV no longer suggested progressive occlusion, but described the left transverse sinus, sigmoid sinus, and internal jugular vein as smaller than the contralateral side (**Figure 1L**, Month 1). Together with no progressive parenchymal injury, improved CSF pleocytosis to approximately 16 cells/μL, and persistently normal D-dimer results, this favored unilateral venous hypoplasia or low-flow change rather than active CVST. The patient was managed with clinical and imaging follow-up rather than escalation of anticoagulation or invasive venous evaluation.

At Month 3, the patient had a clinical relapse characterized by worsening headache and a seizure-like episode with forehead twitching followed by loss of consciousness for approximately 30 minutes, salivation, and urinary incontinence. After the episode, she had transient generalized weakness and slowed speech. Repeat lumbar puncture showed an opening pressure of 200 mmH_2_O, recurrent CSF pleocytosis of approximately 76 cells/μL, and elevated CSF protein of 532 mg/L. During the Month 3 relapse evaluation, repeat CSF pathogen-targeted next-generation sequencing was performed and did not identify a definite pathogen. Because this sample was obtained after approximately 3 months of cumulative antiviral exposure, the negative result was interpreted cautiously and was not used as stand-alone evidence to exclude an earlier viral or post-infectious mechanism. Autoimmune antibody testing during the Month 3 relapse showed CSF anti-NMDAR-IgG positivity and CSF MOG-IgG positivity, both at a titer of 1:3.2 with moderate fluorescence intensity (++). In the paired serum sample obtained at the same time, MOG-IgG was weakly positive at 1:10, whereas serum anti-NMDAR-IgG and other tested neuronal surface or intracellular antibodies, including AMPA1/2, LGI1, CASPR2, GABA_B, DPPX, IgLON5, GAD65, and mGluR5, were negative. This paired CSF/serum antibody profile, interpreted together with the recurrent cortical-sulcal MRI lesions and inflammatory CSF findings, supported the diagnosis of MNOS-associated MOGAD cortical encephalitis rather than an isolated laboratory finding. Autoantibody testing was performed using the same standardized indirect immunofluorescence workflow later detailed in the Laboratory and Diagnostic Methods section, combining tissue-based assay support with fixed cell-based assay readouts for paired CSF and serum samples. Brain MRI at Month 3 demonstrated a new mild linear T2-FLAIR hyperintensity along the right occipital sulci (**Figure 1I**, Month 3), indicating recurrent unilateral cortical/sulcal involvement with laterality different from the earlier left parieto-occipital abnormality. MRA showed no obvious arterial abnormality (**Figure 1M**, Month 3). EEG during the Month 3 relapse showed a mildly abnormal background, with a bilateral symmetric posterior alpha rhythm of 8.5–10.5 Hz, poor modulation, and mildly increased bilateral symmetric theta activity at 4–7.5 Hz. Photic stimulation did not elicit additional abnormalities, and alpha attenuation with eye opening was complete. No epileptiform discharges, electrographic seizures, focal slowing, or extreme delta brush pattern were reported.

Treatment was then redirected toward first-line immunotherapy. The patient received intravenous methylprednisolone pulse therapy, starting at 1,000 mg/day for 3 days, followed by 500 mg/day for 3 days, 250 mg/day for 3 days, and 120 mg/day for 3 days, with subsequent transition to oral corticosteroid tapering. IVIG was administered at 20 g/day for 5 days, and levetiracetam was started for seizure control. No further seizures were documented after immunotherapy. Headache improved, postictal slowing of speech resolved, and the transient weakness recovered before discharge. Urinary incontinence was limited to the ictal episode. During corticosteroid treatment, ACTH suppression and erythematous rash over the face, shoulders, and back were noted and considered steroid-related adverse effects.

Follow-up antibody testing showed a compartment- and time-dependent pattern. Serum MOG-IgG was negative at Month 6, but weakly positive again at 1:10 at Month 11. At Month 14, CSF anti-NMDAR-IgG and CSF MOG-IgG had converted to negative, while serum anti-NMDAR-IgG remained negative and serum MOG-IgG persisted at 1:10. These dynamic, partly compartment-discordant results required interpretation with symptoms, MRI findings, CSF inflammation, and treatment exposure (**Figure 2**).

### Laboratory and diagnostic methods

Autoimmune antibody testing was performed at Unyi Medical Laboratory using a standardized indirect immunofluorescence test (IIFT) workflow read on the EUROStar III platform, combining tissue-based assay (TBA) on mammalian brain sections and fixed cell-based assay (fixed CBA) using antigen-transfected cells. Antibody results were reported according to serial dilution titers and semiquantitative fluorescence grading. According to the laboratory’s IIFT interpretation table, CSF positivity was graded from 1:1 (+, weak), with 1:3.2 corresponding to moderate fluorescence intensity (++), whereas serum positivity was graded from 1:10 (+, weak), with 1:32 corresponding to moderate fluorescence intensity (++). During the Month 3 relapse, paired CSF and serum samples showed CSF anti-NMDAR-IgG positivity and CSF MOG-IgG positivity, both at 1:3.2 (++), together with weak serum MOG-IgG positivity at 1:10. Other tested neuronal surface or intracellular antibodies, including AMPA1/2, LGI1, CASPR2, GABA_B, DPPX, IgLON5, GAD65, and mGluR5, were negative. Follow-up testing was performed using the same laboratory workflow: serum MOG-IgG was negative at Month 6, weakly positive at 1:10 at Month 11, and remained weakly positive at 1:10 at Month 14; at Month 14, CSF anti-NMDAR-IgG and CSF MOG-IgG were both negative, and serum anti-NMDAR-IgG was negative. Because low-positive MOG-IgG results obtained by fixed CBA may have lower positive predictive value and are influenced by sampling timing, low-titer serum MOG-IgG was interpreted together with clinical phenotype, MRI evolution, CSF findings, and treatment exposure rather than as stand-alone evidence of active disease (8).

## Discussion

The significance of this case lies not merely in MOG-IgG and anti-NMDAR-IgG coexistence, but in the longitudinal reassessment needed to distinguish autoimmune cortical encephalitis from initially plausible vascular and infectious alternatives. At presentation, headache, seizure-like symptoms, meningeal signs, CSF inflammation, and unilateral venous sinus non-visualization reasonably prompted consideration of CVST, viral meningoencephalitis, and neuro-Behçet disease. The final diagnosis of MNOS-associated cortical encephalitis within the FLAMES/UCCE spectrum was supported by concordant clinical, radiological, CSF, and antibody findings. Under the 2023 International MOGAD Panel proposed criteria (7), the patient had a compatible core phenotype of cerebral cortical encephalitis, with headache, seizure, inflammatory CSF abnormalities, and unilateral cortical-sulcal T2-FLAIR lesions. Because serum MOG-IgG was only low-positive at 1:10 by fixed cell-based assay, the diagnosis was not based on serology alone, but on supportive MRI evidence: a left parieto-occipital cortical-sulcal lesion at Week 2 and a new contralateral right occipital lesion during the Month 3 relapse. CSF MOG-IgG positivity during relapse further supported the overlap phenotype when interpreted with the clinical, MRI, and CSF findings, while longitudinal reassessment did not support active CVST, infectious meningoencephalitis, or neuro-Behçet disease. The patient also fulfilled the 2016 criteria for definite anti-NMDAR encephalitis (14), with a compatible encephalitic syndrome, seizure with impaired consciousness, speech dysfunction, CSF anti-NMDAR-IgG positivity, and reasonable exclusion of alternative causes.

Radiological lag was an important contributor to the initial diagnostic uncertainty in this case. Recent work on intra-attack MRI dynamics in MOGAD has shown that early brain MRI may be normal despite active cerebral symptoms, with cortical or other T2-FLAIR lesions becoming apparent only on repeat imaging during the same attack (2). This pattern was reflected in our patient: the Day 1 parenchymal MRI showed no definite cortical lesion, whereas follow-up MRI at Week 2 demonstrated a left parieto-occipital cortical-sulcal T2-FLAIR hyperintensity suggestive of FLAMES/UCCE-spectrum cortical involvement. This delay, together with meningoencephalitic symptoms, inflammatory CSF findings, and left transverse-sigmoid sinus/internal jugular venous non-visualization on MRV, increased the plausibility of infectious and vascular alternatives at onset. The subsequent resolution of the left-sided lesion and emergence of a new contralateral cortical-sulcal lesion during relapse further supported a dynamic inflammatory cortical process rather than a fixed venous-territorial lesion. Therefore, a normal early parenchymal MRI should not exclude MOGAD-related cortical encephalitis or MNOS when the clinical and CSF profile remains suspicious; repeat MRI is essential when early imaging and clinical findings are discordant.

At presentation, transient focal symptoms and left transverse-sigmoid sinus/internal jugular vein non-visualization on MRV made CVST a reasonable early consideration. However, the initial parenchymal MRI showed no venous infarction, hemorrhagic venous infarction, mass effect, or other secondary signs of venous hypertension. Because non-contrast MRV can be affected by flow-related signal loss, isolated venous non-visualization should be interpreted with conventional MRI, DWI/ADC, contrast-enhanced venous imaging when available, and follow-up evolution. In this case, serial MRI showed recurrent cortical-sulcal T2-FLAIR lesions with changing laterality rather than a fixed venous-territorial pattern, while the venous abnormality was later described as a small-caliber left-sided venous pathway. Although normal D-dimer alone cannot exclude CVST, the overall clinicoradiological pattern favored venous hypoplasia or low-flow change over active thrombosis (9, 10).

The infectious differential diagnosis also required reassessment. Empirical antiviral therapy was clinically understandable during the first admission because the patient presented with headache, low-grade fever, meningeal signs, marked lymphomononuclear CSF pleocytosis, and epidemiologic exposure to varicella without prior vaccination. Ganciclovir was used because acyclovir was not available locally, but this practical choice did not constitute virological confirmation. The Day 4 CSF DNA-based pathogen sequencing, obtained before antiviral exposure, did not detect a definite DNA pathogen; however, acute CSF HSV/VZV-specific PCR, VZV serology, and intrathecal antibody testing were not performed. Repeat CSF ptNGS at Month 3 was also negative, but the sample was obtained after prolonged antiviral exposure. Therefore, we cannot definitively exclude an earlier herpesvirus-related or post-infectious event. The final diagnosis favored MNOS-associated autoimmune cortical encephalitis because relapse occurred with a contralateral cortical-sulcal lesion, recurrent CSF inflammation, CSF anti-NMDAR-IgG positivity, CSF/serum MOG-IgG positivity, normal MRA, and clinical improvement after corticosteroids and IVIG.

Neuro-Behçet disease was also considered because of recurrent oral ulcers, yet the absence of genital ulcers, uveitis, erythema nodosum-like lesions, and other supportive systemic findings made this diagnosis unlikely (11, 12). Moreover, serial MRI did not show the typical brainstem, diencephalic, or deep parenchymal pattern more commonly associated with parenchymal neuro-Behçet disease. Taken together, these observations shifted the diagnostic framework away from infection, thrombosis, and systemic inflammatory disease toward autoimmune encephalitis.

Consistent with prior reports on anti-NMDAR/MOG overlap and MOG-IgG coexistence with neuronal autoantibodies, this patient showed an encephalitic phenotype characterized by headache, seizures, cortical MRI abnormalities, inflammatory CSF findings, and responsiveness to first-line immunotherapy (1, 4, 13, 15). Prior overlap studies help contextualize this pattern. In the Dalmau-led overlap series, anti-NMDAR encephalitis occurred concurrently with demyelinating syndromes, demonstrating that neuronal and demyelinating autoimmunity may coexist within a single evolving disorder (4). In the Mayo Clinic cohort of MOG-IgG1-positive patients with neuronal autoantibodies, NMDA-R-IgG was the most frequent coexisting neuronal antibody, and the double-positive subgroup was enriched for encephalopathy, seizures, and leptomeningeal enhancement (15). Our patient fit this overlap framework because the dominant phenotype was encephalitic, with headache, seizure, inflammatory CSF abnormalities, and recurrent cortical-sulcal MRI lesions rather than a purely demyelinating presentation. However, beyond these shared features, this case highlights how MNOS may initially enter a CVST-oriented, infection-oriented, or neuro-Behçet-oriented diagnostic pathway when venous imaging abnormalities, meningeal signs, and inflammatory CSF findings coexist. Sequential multimodal reassessment helped redirect the diagnosis from these initially plausible but ultimately unsupported alternatives toward MNOS-associated MOGAD cortical encephalitis within the FLAMES/UCCE spectrum. Therefore, the diagnostic value of dual antibody positivity in this case derived from its concordance with the relapsing encephalitic phenotype, cortical-sulcal MRI evolution, and inflammatory CSF profile, rather than from antibody coexistence alone (4, 7, 14, 15).

A limitation of the infectious evaluation is that no acute CSF HSV/VZV-specific PCR, CSF VZV IgG/IgM, or paired acute/convalescent serology was obtained during the first admission. Although CSF DNA-based high-throughput sequencing before antiviral exposure did not identify a definite DNA pathogen, this result does not fully exclude infection below the assay detection limit or a post-infectious mechanism. This limitation is particularly relevant because MOGAD cortical or meningo-cortical phenotypes can initially mimic infectious meningoencephalitis (3, 5, 6). The transient papular rash was clinically interpreted as a nonspecific viral exanthem, but it did not provide pathogen-specific diagnostic evidence and was insufficient to establish a direct causal link to the encephalitic syndrome.

A significant radiological limitation of the clinical workup in this case is that gadolinium-contrast-enhanced T1-weighted or contrast-enhanced fluid-attenuated inversion recovery (CE-FLAIR) sequences were not performed during the active phases of cortical inflammation (specifically at Week 2 and Month 3). Cortical and overlying leptomeningeal enhancement (LME) represents a diagnostic hallmark of MOGAD-associated meningocortical syndromes, including the FLAMES and UCCE spectrum, reflecting active perivascular and leptomeningeal inflammatory infiltration with localized blood-brain barrier disruption. Although the diagnosis in our patient was successfully established based on the sequential development of unilateral cortical-sulcal T2-FLAIR lesions with shifting laterality, recurrent inflammatory CSF profiles, and paired CSF/serum double-antibody positivity, the lack of contrast-enhanced imaging precluded direct visualization of this characteristic meningeal inflammatory activity. Recent pediatric studies have demonstrated that CE-FLAIR imaging is more sensitive than conventional contrast-enhanced T1-weighted imaging for detecting leptomeningeal enhancement in MOGAD, and that isolated LME without parenchymal T2-FLAIR abnormalities may represent the sole radiological manifestation of meningocortical MOGAD in some cases (17, 18). In clinical practice, contrast-enhanced neuroimaging is highly recommended in patients suspected of having cortical encephalitis to definitively exclude infectious or neoplastic leptomeningitis, fully delineate the extent of leptomeningeal involvement, and assess disease activity during follow-up.

The antibody profile required phase- and compartment-specific interpretation. CSF anti-NMDAR-IgG was diagnostically relevant in a compatible encephalitic syndrome (14), whereas MOG-IgG supported MOGAD only when concordant with the clinical and MRI phenotype (7). In this patient, the coexistence of CSF anti-NMDAR-IgG and CSF/serum MOG-IgG during relapse supported a true overlap syndrome because it coincided with recurrent cortical-sulcal MRI abnormalities and inflammatory CSF findings. During follow-up, however, serum MOG-IgG became negative at Month 6 and later returned to weak positivity at 1:10, whereas CSF anti-NMDAR-IgG and CSF MOG-IgG were negative at Month 14. Persistent or recurrent serum MOG-IgG may support concern for a relapsing-prone course at the cohort level, consistent with the relapse observed in this patient (16); nevertheless, a single weak-positive serum result should not be used as stand-alone evidence of active inflammatory disease or as the sole basis for treatment escalation (7, 8). Follow-up assessment should integrate symptoms, serial MRI, CSF inflammation, sampling compartment, assay type, and treatment exposure.

### Key clinical takeaways

1. Pediatric MNOS can mimic CVST or infectious meningoencephalitis when headache, meningeal signs, CSF inflammation, and nondiagnostic early MRI coexist.
2. MRV venous non-visualization does not prove active CVST; diagnosis should integrate D-dimer, serial imaging, and clinicoradiological concordance (9, 10).
3. Persistent or recurrent MOG-IgG may indicate relapse risk, but weak serum positivity alone should not define relapse without clinical, MRI, or CSF support.

## Patient perspective

At the beginning of the illness, I was mainly troubled by persistent headache and uncertainty about the diagnosis. When I later experienced an episode with loss of consciousness, my family and I became very frightened. After immunotherapy, my symptoms gradually improved, and I became more confident about returning to school and normal daily activities.

## Data Availability

The original contributions presented in the study are included in the article/supplementary material. Further inquiries can be directed to the corresponding author.
